# Nanoenabled Intracellular Metal Ion Homeostasis Regulation for Tumor Therapy

**DOI:** 10.1002/advs.202306203

**Published:** 2023-12-08

**Authors:** Lihua Xu, Mingzheng Peng, Tingting Gao, Dandan Wang, Xiaowu Lian, Huihui Sun, Jinjin Shi, Yafeng Wang, Pengju Wang

**Affiliations:** ^1^ Sino‐British Research Centre for Molecular Oncology National Centre for International Research in Cell and Gene Therapy State Key Laboratory of Esophageal Cancer Prevention & Treatment School of Basic Medical Sciences Academy of Medical Sciences Zhengzhou University Zhengzhou 450052 China; ^2^ School of Pharmaceutical Sciences Zhengzhou University Zhengzhou 450001 China; ^3^ Henan Institute of Medical and Pharmaceutical Sciences Zhengzhou University Zhengzhou 450052 China

**Keywords:** antitumor mechanism, intracellular metal ion homeostasis regulation, nanomedicine, synergistic therapy, tumor immunity

## Abstract

Endogenous essential metal ions play an important role in many life processes, especially in tumor development and immune response. The approval of various metallodrugs for tumor therapy brings more attention to the antitumor effect of metal ions. With the deepening understanding of the regulation mechanisms of metal ion homeostasis in vivo, breaking intracellular metal ion homeostasis becomes a new means to inhibit the proliferation of tumor cells and activate antitumor immune response. Diverse nanomedicines with the loading of small molecular ion regulators or metal ions have been developed to disrupt metal ion homeostasis in tumor cells, with higher safety and efficiency than free small molecular ion regulators or metal compounds. This comprehensive review focuses on the latest progress of various intracellular metal ion homeostasis regulation‐based nanomedicines in tumor therapy including calcium ion (Ca^2+^), ferrous ion (Fe^2+^), cuprous ion (Cu^+^), managanese ion (Mn^2+^), and zinc ion (Zn^2+^). The physiological functions and homeostasis regulation processes of ions are summarized to guide the design of metal ion regulation‐based nanomedicines. Then the antitumor mechanisms of various ions‐based nanomedicines and some efficient synergistic therapies are highlighted. Finally, the challenges and future developments of ion regulation‐based antitumor therapy are also discussed, hoping to provide a reference for finding more effective metal ions and synergistic therapies.

## Graphical abstract

This review focuses on the application of nanoenabled intracellular metal ion homeostasis regulation in tumor therapy including calcium ion (Ca^2+^), ferrous ion (Fe^2+^), cuprous ion (Cu^+^), managanese ion (Mn^2+^), and zinc ion (Zn^2+^). The physicochemical properties, physiological functions, and homeostasis regulation processes of the ions are summarized, and the antitumor mechanisms of various ions‐based nanomedicines at both cell‐ and tissue‐level and some efficient synergistic therapies are highlighted.

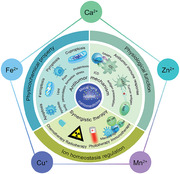

## 1 Introduction

Cancer is one of the leading causes of mortality worldwide, with an increasing social and financial burden. Traditional therapies in the clinic include surgery, chemotherapy, radiotherapy, phototherapy, thermal therapy, and immunotherapy, in which chemotherapy is still the first choice for most patients. Cisplatin (*cis*‐diaminodichloroplatinum, CDDP) is the first‐line platinum (Pt)‐containing chemotherapeutic agent applied in the treatment of human malignant tumors, such as lung, breast, and ovarian. The antitumor mechanism of Pt‐containing drugs is mainly associated with the formation of Pt‐DNA adducts and the inhibition of DNA, RNA, and protein synthesis in tumor cells.^[^ ^1^ ^]^ The deep research on antitumor mechanisms and the structure‐activity relationship of anticancer activity make platinum drugs more efficient and less toxic. Besides Pt‐containing metallodrugs, arsenic (As)‐based drugs (e.g., As_2_O_3_) are also widely used in clinics for acute promyelocytic leukemia (APL) and primary liver cancer. In addition, argentum (Ag)‐based metallodrugs (e.g., Ag nanoparticles) have been widely used as antimicrobial agents and suggested to exhibit antitumor effects from many preclinical research results.^[^ ^2^ ^]^ Auranofin is an approved gold‐based drug for rheumatoid arthritis and has been demonstrated to possess antitumor activity. Excitingly, a few ruthenium‐based antitumor drugs (e.g., NAMI‐A, KP1019, TLD1433) are under clinical research.

Inspired by the efficient antitumor effects of metallodrugs via the introduction of exogenous metal ions, the role of endogenous essential metals (e.g., calcium, iron, zinc, copper, and manganese) in tumor treatment has gained much attention in recent years. It is known that various essential metal ions exist in the human body and participate in many life activities, such as signal pathway activation, enzyme catalysis and protein composition, etc.^[^ ^3^ ^]^ The abnormal distribution or specific accumulation of some metal ions can activate cytotoxicity‐related biochemical reactions to induce cell death.^[^ ^4^ ^]^ In normal cells, ion homeostasis is tightly and precisely regulated for cell survival, metabolism, and immunity.^[^ ^5^ ^]^ However, due to the properties of immortalization and immune escape of tumor cells, intracellular metal ions‐related signal pathways (e.g., calcium ion (Ca^2+^) signal) are remodeled for survival, leading to the sensitivity to intracellular metal ion regulation‐mediated tumor therapy.^[^ ^6^ ^]^ In addition, with the deepening understanding of the function of metal ions in cancer immunotherapy, a new term, “metalloimmunology”, was proposed in 2020, and “cancer metalloimmunotherapy” was subsequently described in 2021, indicating that metal ions also play an important role in tumor immunoregulation.^[^ ^7^ ^]^

Metal ion regulation‐mediated tumor therapy is an emerging approach that depends on direct or indirect alterations of intracellular metal ion concentrations for tumor inhibition and immunoregulation.^[^ ^8^ ^]^ Many small molecular compounds or metallic compounds are employed for the regulation of ion regulation‐related proteins or intracellular free metal ions. However, obstacles, such as poor specific recognition ability and insufficient changes in ion concentration limit their effect on cancer treatment.^[^ ^9^ ^]^ Metal ions‐containing nanomedicines can not only efficiently deliver small molecular drugs to tumor cells due to the advantages of tumor‐targeting capability and protection of drug activity, but also directly induce irreversible ions overload or other mechanisms for tumor therapy, like self‐therapeutic agents.^[^ ^10^ ^]^ For example, Rizzolio's group^[^ ^11^ ^]^ demonstrated that metal‐based nanomaterials like silver nitroprusside nanoparticles, and iron nitroprusside nanoparticles were efficiently self‐therapeutic for tumors. So, metal ions regulation‐based nanomedicines have great potential for tumor therapy.

**Figure 1:**
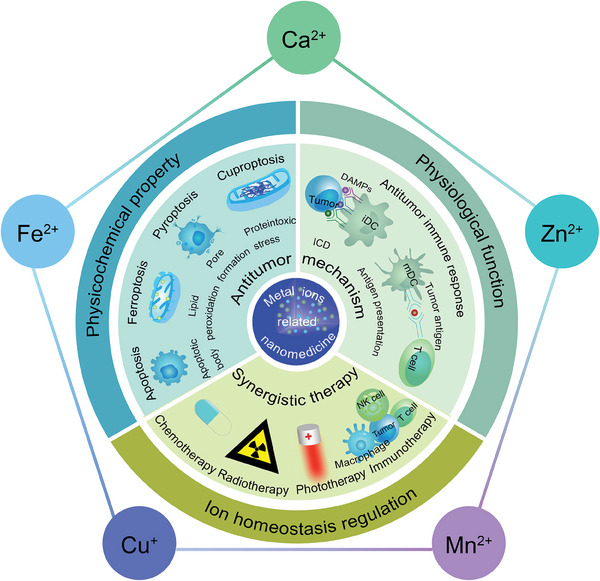
Schematic overview of metal ions‐related nanomedicines for tumor therapy.

This review focuses on the application of the five attractive metal ions, including Ca^2+^, ferrous ion (Fe^2+^), cuprous ion (Cu^+^), manganese ion (Mn^2+^), and zinc ion (Zn^2+^) in tumor therapy (**Figure** **1**). The physicochemical properties, physiological functions, and regulation mechanisms of intracellular ion homeostasis about the five ions are first discussed. In addition, antitumor mechanisms of ion regulation‐related nanomedicines and potential synergistic treatment strategies are highlighted. The future prospects and challenges of intracellular metal ion regulation‐based nanomedicines are also explored in order to promote further research and application in tumor therapy.

## 2 Characteristics of Ca^2+^

### 2.1 Physicochemical Properties and Physiological Functions of Calcium

Calcium (element symbol: Ca) is a chemical element with the atomic number 20 and its ionic form is Ca^2+^. Ca is the most abundant metal element in humans and essential in skeletons and teeth. Free Ca^2+^, Ca composite, and protein binding Ca are the three main forms of Ca, which interconvert with each other. However, free Ca^2+^ is the only form that possesses physiological activity. As the second messenger, Ca^2+^ is involved in diverse cellular progress, such as proliferation, multidrug resistance, migration, death, and immune response.^[^ ^12^ ^]^ In the cell, the concentration of free Ca^2+^ is only 100 nm, which is 10 000 times lower than the extracellular calcium concentration.^[^ ^13^ ^]^ Fluctuations in Ca^2+^ concentration interfere with the transmission of normal calcium signals and thus affect the normal physiological function of cells. The endoplasmic reticulum (ER), mitochondria, and sarcoplasmic reticulum are three main Ca^2+^ stores and play an essential role in maintaining intracellular Ca^2+^ homeostasis.^[^ ^14^ ^]^

Given the importance of Ca^2+^ homeostasis for cell survival, there are complex regulatory processes and many vital molecules involved in Ca^2+^ transport to maintain the intracellular Ca^2+^ concentration ([Ca^2+^]_i_) within a certain range. Ca^2+^‐related regulators consist of Ca^2+^ channels, Ca^2+^‐activated ATPases, and exchangers.^[^ ^15^ ^]^ Ca^2+^ channels are located in the cytomembrane (e.g., calcium release‐activated calcium modulator 1 (ORAI1) channel), ER membrane (e.g., inositol 1,4,5‐trisphosphate receptors (IP3Rs) and ryanodine receptors (RyRs)) or mitochondrial inner membrane (e.g., mitochondrial Ca^2+^ uniporter (MCU), devoting to extracellular Ca^2+^ influx, ER Ca^2+^ refilling or mitochondrial Ca^2+^ filling.^[^ ^16^ ^]^ Ca^2+^ channels are either voltage‐dependent or receptor‐operated, not adenosine triphosphate (ATP)‐dependent, while Ca^2+^ activated ATPases (e.g., plasma membrane calcium pumps (PMCAs)), and exchangers (e.g., Na^+^/Ca^2+^ exchangers) contributed to ATP‐dependent Ca^2+^ flux.^[^ ^17^ ^]^

Herein, we take the case of intracellular Ca^2+^ overload as an example to elucidate the regulation processes of intracellular Ca^2+^. To maintain Ca^2+^ homeostasis, PMCAs and Na^+^/Ca^2+^ exchangers in the cell membrane will pump excess free cytoplasmic Ca^2+^ out of the cell first, and the Ca^2+^ channels in the cell membrane are closed. In addition, partial Ca^2+^ is actively transported into the ER or mitochondria by sarco/endoplasmic reticulum Ca^2+^ ATPase (SERCA) pumps or mitochondrial calcium uniporter regulators.^[^ ^18^ ^]^

### 2.2 Antitumor Mechanisms of Ca^2+^ Overload‐Based Nanomedicines

In normal cells, regulation of intracellular Ca^2+^ flux is operated by an interconnected machinery at multiple organelles, allowing Ca^2+^ ions to act as second messengers meanwhile preventing their potential cytotoxicity. Different from normal cells, Ca^2+^ signaling pathways are remodeled in tumor cells, contributing to tumor hallmarks such as aberrant proliferation, apoptosis resistance, and metastatic dissemination. Compared with that in normal cells, dysregulation and abnormal expression of several Ca^2+^‐activated ATPases, channels, or calcium‐binding proteins are characteristics that are exclusive to many cancers, indicating that tumor cells are more sensitive to Ca^2+^ regulation than normal cells.^[^ ^19^ ^]^ Calcium dyshomeostasis comprises Ca^2+^ exhaustion and Ca^2+^ overload. Given the high concentration of extracellular Ca^2+^, Ca^2+^ exhaustion by Ca^2+^ antagonists (e.g., verapamil) shows limited antitumor efficacy.^[^ ^20^ ^]^

**Figure 2:**
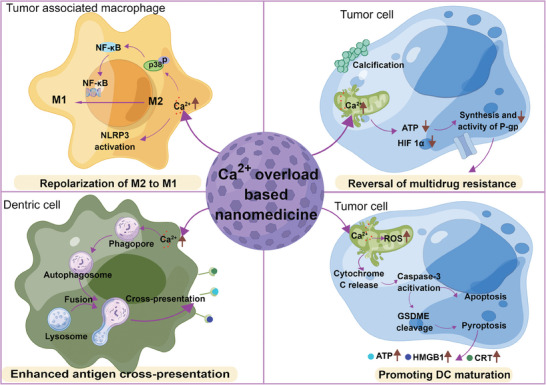
Schematic diagram of antitumor mechanisms of Ca^2+^ overload‐based nanomedicines.

In recent years, the Ca^2+^ overload‐based strategy has become a research hotspot for anti‐tumor therapy. Excessive intracellular free Ca^2+^ is confirmed to induce tumor cell death.^[^ ^21^ ^]^ To date, small molecular drugs, calcium electroporation, and Ca^2+^‐containing nanomaterials are three main approaches to cause intracellular Ca^2+^ overload. The mechanism of most small molecular drugs (e.g., curcumin (CUR)) is to act on Ca^2+^‐related signaling pathways without the addition of exogenous Ca^2+^, while calcium ionophores (e.g., ionomycin) can introduce exogenous Ca^2+^ into cytoplasm.^[^ ^22^ ^]^ Calcium electroporation is the combination of intratumoral injection of calcium with local high‐frequency electrical pulses.^[^ ^23^ ^]^ With the development of nanotechnology, calcium‐based nanomaterials are used to directly and effectively elevate the intracellular Ca^2+^ concentration. What's more, calcium‐based nanocarriers are among the safest materials owing to good biocompatibility and pH‐sensitive biodegradability. For example, calcium carbonate nanoparticles have been used for bone repair or Ca supplementation in medical or food fields. Calcium phosphates (CaPs), calcium carbonates (CaCs), calcium silicate (CaSi), and calcium fluoride (CaF_2_) are the main calcium‐based nanomaterials commonly used in tumor therapy.^[^ ^24^ ^]^ To date, the antitumor mechanisms of Ca^2+^ overload‐based nanomedicines consist of mitochondrial dysfunction‐mediated apoptosis, inhibition of efflux pumps, pyroptosis, and remodeling of immunosuppressive microenvironment (**Figure** **2**). In this section, we will discuss the detailed antitumor mechanisms that have been reported by calcium‐based nanomedicines and possible synergistic therapies in recent years.

#### 2.2.1 Mitochondrial Dysfunction‐Mediated Apoptosis

As a vital type of subcellular organelles, mitochondria not only provide ATP for the activities of the cell, but are implicated in various signaling pathways, e.g., cellular proliferation, apoptosis, and calcium dynamics.^[^ ^25^ ^]^ Mitochondria control the uptake and release of Ca^2+^ through channels/transporters, such as MCUs, and influence the concentration of Ca^2+^ in both mitochondria and cytoplasm, thereby regulating cellular Ca^2+^ homeostasis. Multiple studies have revealed that mitochondrial Ca^2+^ overload is a promising approach to tumor cell apoptosis. The main proapoptotic mechanisms are as follows: 1) Mitochondrial Ca^2+^ overload can activate Ca^2+^‐dependent phospholipases and endonucleases, leading to cellular and organelle membrane damage and cytoskeletal destruction. 2) Ca^2+^ overload in the mitochondria effectively disturbs the mitochondrial membrane potential and blocks ATP production by deposition of Ca_3_(PO_4_)_2_. 3) Dysfunctional mitochondria release apoptotic factors (e.g., cytochrome C) through the opened mitochondrial permeability pore, followed by activating the caspase‐3‐related apoptosis pathway. 4) Mitochondrial Ca^2+^ overload can increase intracellular reactive oxygen species (ROS) levels, further promoting apoptosis.^[^ ^26^ ^]^

Ca^2+^ overload‐based nanomedicines include Ca^2+^ contained nanomaterials, or small molecule drugs loaded two‐in‐one nanomedicines. Research from Bu's group^[^ ^27^ ^]^ demonstrated that CaO_2_ nanoparticles could result in intracellular ROS generation and Ca^2+^ overload, inducing calcification and apoptosis of tumor cells. Sun et al.^[^ ^28^ ^]^ found that HAP nanoparticles triggered efficient mitochondrial targeting and a sustained elevation of [Ca^2+^]_i_, thus inducing mitochondria‐dependent apoptosis. Moreover, Chang and colleagues^[^ ^29^ ^]^ designed and engineered a distinct valence‐invariable CaF_2_ nanozyme with ultrasound (US)‐enhanced peroxidase (POD)‐mimicking activity. Exogenous Ca^2+^ ions introduction and calcium‐pumping channel regulation by CaF_2_ nanocrystals significantly achieved calcium overload‐induced mitochondrial dysfunction and apoptosis.

To further boost Ca^2+^ overload‐mediated antitumor therapy, Li and colleagues developed a kaempferol‐3‐O‐rutinoside (KAE) loaded CaCO_3_ nanomedicine as a two‐in‐one Ca^2+^ overload‐based nanomedicine.^[^ ^30^ ^]^ Ca^2+^ influx by KAE and Ca^2+^ supply by CaCO_3_ made the nanomedicine become a calcium bomb, which destructed mitochondrial structure and functions, causing cytoskeleton collapse and oxidative stress and leading to cancerous cellular apoptosis. Our previous study also confirmed the concept by combining CaP‐dopped hollow mesoporous copper sulfide (HMCuS) nanoparticles (a Ca^2+^ nanogenerator) with a small molecule CUR which inhibited Ca^2+^ efflux and facilitated ER Ca^2+^ release.^[^ ^31^ ^]^ In further research, Wang et al.^[^ ^32^ ^]^ delivered CUR by amorphous CaCO_3_ instead of CaP, achieving a sustained Ca^2+^ overload and enhanced tumor cell apoptosis as well.

Taken together, Ca^2+^ overload‐based nanomedicines contribute to mitochondrial Ca^2+^ overload‐mediated apoptosis with intracellular ATP production blockade.

#### 2.2.2 Reversal of Multidrug Resistance by Inhibition of Efflux Pumps

Multidrug resistance (MDR) is a major contributor to the failure of tumor therapy, and overexpression of efflux pumps on the cell membrane is one of the most important reasons for MDR. Efflux pumps mainly consist of ATP‐binding cassette (ABC) transporters such as P‐glycoprotein (P‐gp). Studies have reported that P‐gp works as a regulator by promoting the efflux of diverse drugs in tumor cells in an ATP‐dependent manner, and expression and activity of P‐gp are considered as two important targets.^[^ ^33^ ^]^ Delivering small molecule inhibitors or small interfering RNA of P‐gp to tumor cells has been used to effectively reverse drug resistance by inhibiting P‐gp activity.^[^ ^34^ ^]^ In addition, cellular internalization and excretion of nanomaterials through endocytosis and exocytosis pathways have enabled them to bypass drug efflux pumps, and directly deliver drugs to subcellular organelles.^[^ ^35^ ^]^

To avoid drug efflux, Qian and colleagues confirmed that HAP nanoparticles could efficiently reverse the resistance of MCF‐7/ADR to doxorubicin (DOX) via enhanced delivery and retention of DOX and inhibition of P‐gp activity.^[^ ^36^ ^]^ In addition, our previous study revealed the detailed effects of Ca^2+^ nanomodulators on ABC transporters using CaP nanoparticles as an example.^[^ ^37^ ^]^ We confirmed that CaP nanoparticles could not only inhibit P‐gp biosynthesis by downregulating hypoxia‐inducible factor 1α (HIF 1α) expression but suppress its efflux function by blocking intracellular ATP production.

Overall, on the one hand, acting as conventional nanocarriers, Ca^2+^ overload‐based nanomedicines can facilitate the cellular uptake and retention of drugs. On the other hand, as bioactive nanocarriers, they can inhibit biosynthesis and activity of P‐gp by mitochondrial Ca^2+^ overload.

#### 2.2.3 Boosting Immunotherapy by Pyroptosis and Tumor Immunosuppressive Microenvironment Remodeling

Distinct from apoptosis, pyroptosis is recognized as a process of inflammation‐dependent and gasdermin‐mediated nonapoptotic programmed cell death (PCD), characterized by pore formation, cellular swelling with large bubbles, and leakage of cell content including pro‐inflammatory molecules.^[^ ^38^ ^]^ With the stimulation of extracellular or intracellular inflammatory signals, caspase‐1 is activated to recognize and cleave gasdermin D (GSDMD) to GSDMD‐N and GSDMD‐C domains. Subsequently, GSDMD‐N combines with membrane phospholipid and induces pore formation, resulting in cell swelling with large bubbles. Meanwhile, inflammatory molecules, such as interleukin‐1β (IL‐1β) and interleukin‐18 (IL‐18), can trigger strong inflammation. Besides the classical pathways, studies have reported that the activated caspase‐4/5/11 and activated caspase‐3 in the presence of gasdermin E (GSDME) protein can also effectively induce the occurrence of pyroptosis.^[^ ^39^ ^]^ What's more, Ansara's group and Liu's group jointly showed that pyroptosis not only effectively killed tumor cells, but also caused strong immunogenic cell death (ICD) to produce anti‐tumor immune activity via the release of damage‐related molecular patterns (DAMPs, e.g., ATP, calreticulin (CRT) and high mobility group protein B1 (HMGB1)), which were published in *Nature*.^[^ ^40^ ^]^

Inspired by mitochondrial Ca^2+^ overload‐mediated caspase‐3 activation via Ca^2+^ nanomedicines and the mechanism of pyroptosis, Zheng et al.^[^ ^41^ ^]^ developed a biodegradable Ca^2+^ nanomodulator (CaNM) that can activate pyroptosis for cancer immunotherapy. They proved that mitochondrial Ca^2+^ overload induced by CaCO_3_ nanoparticles and curcumin could contribute to pyroptosis via ROS/caspase‐3/GSDME pathway for the first time. At the same time, they demonstrated that during pyroptosis induced by CaNMs, the released inflammatory molecules and cellular contents, including lactate dehydrogenase (LDH), ATP, and CRT could promote dendritic cells (DC) maturation, causing robust immune responses and tumor metastasis inhibition.

Furthermore, Shi's group^[^ ^42^ ^]^ investigated the effect of Ca^2+^ nanomedicines on immune‐related cells. They found that besides increasing the DAMPs, Ca^2+^‐based nanoparticles could also reset tumor‐associated M2 macrophages to M1 phenotype and induce DC autophagy‐mediated antigen cross‐presentation enhancement for remodeling tumor microenvironment (TME) and boosting chemotherapy and immunotherapy.

Collectively, Ca^2+^ overload‐based nanomedicines can augment tumor immunotherapy by the release of DAMPs and regulation of immune‐related cells in the tumor microenvironment.

## 3 Characteristics of Fe^2+^

### 3.1 Physicochemical Properties and Physiological Functions of Iron

Iron (element symbol: Fe) is the 26th metal element in the periodic table, and its valence states comprise 0, +2, +3, and +6, among which Fe^2+^ and Fe^3+^ are the most common and interconvertible ionic forms in human beings. As a kind of typical transition metal, Fe‐containing compounds usually possess good redox properties, some of which own superparamagnetism such as Fe_3_O_4_.^[^ ^43^ ^]^ Inside the human body, Fe is an important essential trace element, mainly in the form of heme iron (organic iron) and non‐heme iron (inorganic iron). Heme irons are the essential component of hemoglobin in red blood cells and myoglobin in muscles that are responsible for oxygen transport in blood and oxygen storage in muscles, respectively.^[^ ^44^ ^]^ In contrast to heme iron, non‐heme iron is acknowledged to coexist with iron‐binding proteins, such as ferritin, hemosiderin, and neuromelanin.^[^ ^45^ ^]^

Besides the important role in hemoglobin synthesis and oxygen transport when combined with porphyrin, iron is involved in many important cellular processes, such as DNA synthesis and repair, electron transfer, and respiration as a cofactor of various enzymes.^[^ ^46^ ^]^ Maintaining iron homeostasis is crucial for general health and cell viability. Iron deficiency or iron overload are both detrimental, leading to iron‐related diseases. In the human body, iron homeostasis is tightly regulated with iron concentrations in the blood plasma in the range of ≈10–30 µm.^[^ ^47^ ^]^ The small intestine is the only site of iron absorption, which relies on the four proteins of iron metabolism, including duodenal cytochrome b (Dcytb), divalent metal transporter 1 (DMT1), hephaestin (Hp), and ferroportin 1(FPN1).^[^ ^48^ ^]^ The liver is one of the main organs for iron storage in the form of ferritin. It can be seen from the reported studies that FPN1 is the only protein located at the cell surface for transporting iron, and hepcidin is the essential regulator for iron metabolism by interacting with FPN1.^[^ ^49^ ^]^

Inside the cells, iron homeostasis is precisely regulated by iron regulatory proteins (IRPs) and iron‐responsive elements (IREs) at both transcriptional and translational levels, aimed at controlling the iron uptake, storage, and efflux as well as the intracellular iron management and distribution.^[^ ^50^ ^]^ Hepcidin regulates the iron absorption rate while intracellular iron is pumped by bounding to FPN1. Ferritin works as a labile iron pool (LIP) by binding and sequestering intracellular iron. Under physiological circumstances, Fe^3+^ bounding to transferrin (Tf) enters into endosomes by transferin receptors (TfR)‐mediated endocytosis. Free Fe^3+^ dissociated from Tf is reduced to Fe^2+^ by ferrireductases and transported to the cytoplasm by DMT1. In the cell, Fe^2+^ is the major active form by acting as structural or catalytic cofactors of various enzymes and initiating redox reaction. Once iron overload occurs, excessive Fe^2+^ has the capacity to enhance redox cycling and free radical formation via the Fenton reaction, contributing to oxidative stress and initiating signals that are critical to cell survival and cell death.^[^ ^51^ ^]^

### 3.2 Antitumor Mechanisms of Fe^2+^ Dyshomeostasis‐Based Nanomedicines

Contrary to normal cells, iron accumulation occurs by reprogramming intracellular iron metabolism in tumor cells such as downregulated expression of FPN, which is essential for tumorigenesis and development.^[^ ^52^ ^]^ Abundant iron in tumor cells acts as a structural or catalytic cofactor of various proteins, and excess ions are stored in the ferritin, limiting the toxicity. Therefore, targeting iron‐associated proteins, or directly altering intracellular iron levels, is considered a viable strategy for tumor therapy. Downregulation of intracellular free Fe^2+^ by iron chelators delivering nanocarriers is the direct way to inhibit the iron‐associated protein synthesis and signal pathways regulation, which are necessary for tumor survival. In addition, the upregulation of intracellular free Fe^2+^ by iron‐associated protein targeting nanomedicines or iron‐containing nanoparticles is a feasible approach to amplify the toxicity of Fe^2+^ involved in the Fenton reaction. The occurrence of the Fenton reaction can not only kill tumor cells, but also remodel the TME. In this section, we will summarize the detailed antitumor mechanisms of Fe^2+^ dyshomeostasis‐based nanomedicines and possible synergistic therapies.

#### 3.2.1 Downregulation of Intracellular Fe^2+^ for Inhibition of Tumor Growth

**Figure 3:**
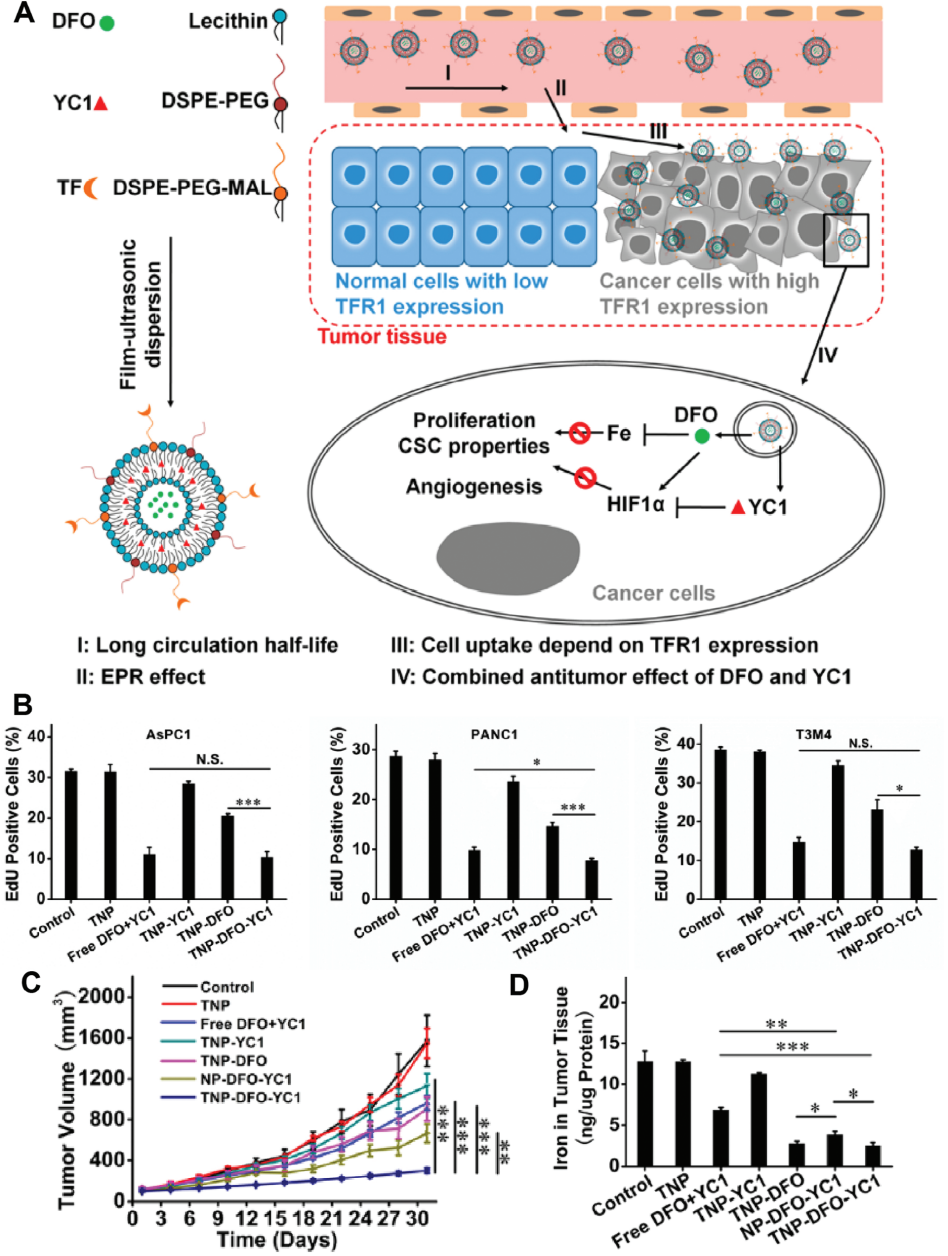
Fe^2+^ chelating mediated antitumor therapy. A) Schematic diagram of synthesis and antitumor mechanism of the nanomedicine co‐delivering DFO and HIF1α inhibitor YC1. B) Cell proliferation, assessed using EdU incorporation, following treatment of pancreatic cell lines. C) Tumor volume changes and D) Iron in tumor tissues with different treatments. Reproduced with permission.^[^ ^54^ ^]^ Copyright 2019, American Chemical Society.

Due to the important role of Fe^2+^ in tumor growth and metastasis, downregulation of intracellular Fe^2+^ was once considered an effective way to inhibit tumor growth. Desferrioxamine (DFO), a widely used iron chelator, has been applied in iron overload‐related diseases. Studies showed that DFO can also exert antitumor activity mainly by depriving essential iron from iron‐dependent enzymes.^[^ ^53^ ^]^ With the advantages of tumor targeting and the high loading efficiency of nanoparticles, DFO‐loaded nanomedicines were designed to enhance the antitumor activity of DFO. For example, Nie's group designed a DFO and YC1 (a HIF1α inhibitor) codelivery nanomedicine to enhance the antitumor activity of DFO by reducing the HIF1α overexpression induced by DFO (**Figure** **3**A).^[^ ^54^ ^]^ The results showed that the codelivery nanomedicine exerted a similar antitumor effect in vitro but better in vivo compared with a free combination of DFO and YC1 (Figure 3B,C). The designed nanomedicine presented the strongest Fe‐chelating ability in vivo (Figure 3D).

In addition, Nishiyama and colleagues^[^ ^55^ ^]^ found that the good iron‐chelating ability of the polymeric DFO nanomedicine led to inhibition of DNA synthesis, cell cycle arrest, and apoptosis. Given that Fe^2+^ is reported as the cofactor of DNA repair enzymes (e.g., alkylation repair homolog 2 (ALKBH2)), our previous studies proved that downregulation of intracellular Fe^2+^ can sensitize DNA damage‐related therapy (e.g., CDDP chemotherapy, and 5‐aminolevulinicacid (5‐ALA) phototherapy).^[^ ^56^ ^]^

#### 3.2.2 Fenton Reaction‐Mediated Ferroptosis and Remodeling of TME by Fe^2+^ Overload

As a transition metal, iron possesses good redox properties. Since the concentration of hydrogen peroxide (H_2_O_2_) in tumor cells is relatively higher than that in normal cells, excess labile iron can trigger a Fenton reaction with H_2_O_2_, which was first proposed by Haber and Weiss in 1934.^[^ ^57^ ^]^ In the Fenton reaction, Fe^2+^ donates an electron to H_2_O_2_ to produce hydroxyl radicals (▪OH). As the most toxic of ROS, ▪OH can destroy biological molecules such as lipids, proteins, and DNA, inducing tumor cell death. The Fenton reaction can be given in Equation (1)

(1) $${\mathrm{Fe}}^{2+}+H_{2}O_{2}\to {\mathrm{Fe}}^{3+}+•\mathrm{OH}+{\mathrm{OH}}^{-}$$

Ferroptosis is a type of programmed and iron‐dependent cell death driven by lipid peroxides (LPO) accumulation.^[^ ^58^ ^]^ Because of the higher intracellular iron capacity and tumor microenvironment, tumor cells are more susceptible to ferroptosis inducers than their normal counterparts.^[^ ^59^ ^]^ As mentioned above, the generated ▪OH in the Fenton reaction can induce cellular lipid peroxidation thereby inducing cellular ferroptosis. Xiong et al.^[^ ^60^ ^]^ constructed a self‐assembled nano‐activator that can hijack endogenous iron in lysosomes to induce ferroptosis. However, insufficient intracellular free iron levels severely hinder the effect of ferroptosis. Therefore, elevating intracellular free Fe^2+^ level is a feasible way for tumor treatment via the Fenton reaction‐mediated ferroptosis.

**Figure 4:**
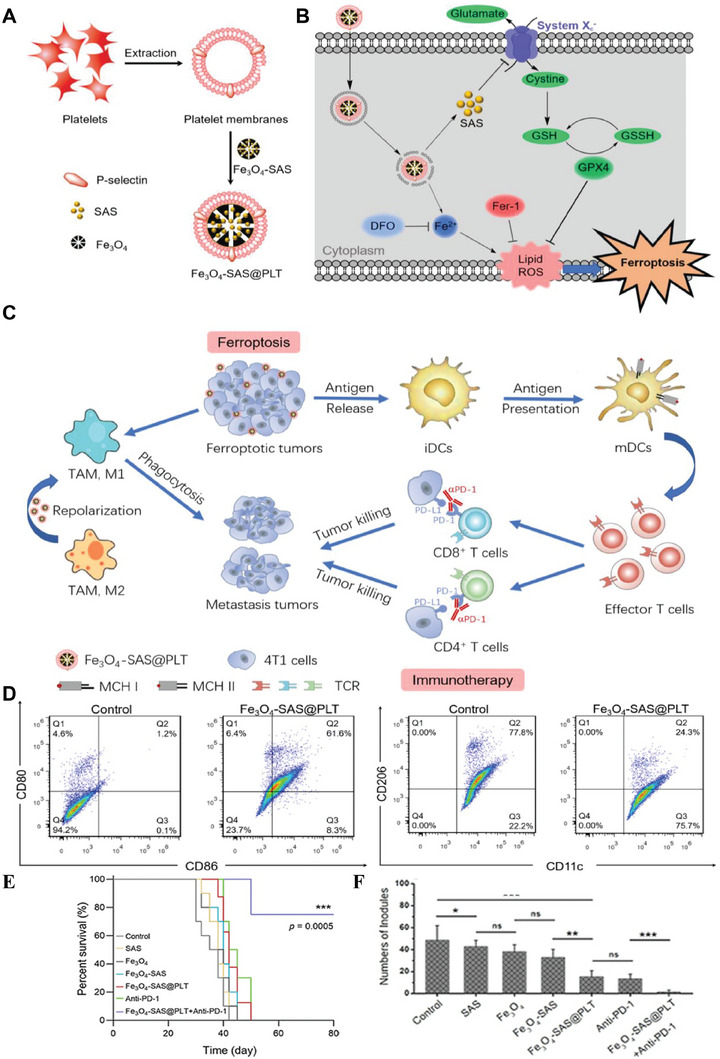
Schematic illustrations of ferroptosis‐mediated antitumor immune responses. A) Synthetic process of Fe_3_O_4_‐SAS@PLT nanoparticles. B) Scheme of ferroptosis mechanisms of Fe_3_O_4_‐SAS@PLT nanoparticles. C) Scheme of regulation of tumor microenvironment by Fe_3_O_4_‐SAS@PLT nanoparticles. D) Representative flow cytometric analysis of CD86^+^/CD80^+^ cells gated on CD11c^+^ cells (left) and CD11c^+^CD206^+^ cells (right). E) Survival analysis of mice treated with different formulas. F) The number of lung metastasis nodules. Reproduced with permission.^[^ ^63^ ^]^ Copyright 2020, WILEY‐VCH Verlag GmbH & Co. KGaA, Weinheim.

With the development of nanomaterials, nanomedicines that contain Fe^2+^/Fe^3+^ or deliver Fe^2+^/Fe^3+^ compounds have been developed for inducing ferroptosis. Diverse nanocarriers such as polydopamine (PDA) and amorphous CaCO_3_ nanoparticles have been used for Fe^2+^ delivery.^[^ ^61^ ^]^ Due to the limited H_2_O_2_ in tumor cells, Fe^2+^‐mediated ferroptosis can be boosted by chemotherapeutics (e.g., cisplatin) via H_2_O_2_ concentration elevation.^[^ ^62^ ^]^ Fe_3_O_4_ nanoparticles are a widely used and relatively safe ferroptosis inducer, because it has been used for clinical research, such as iron deficiency therapy, magnetic resonance imaging contrast agent, or drug carrier. In addition, iron‐containing nanoparticles (e.g., Fe_3_O_4_ nanoparticles) can not only release Fe^2+^ in the acidic lysosome, but also deliver antitumor drugs to synergistically induce ferroptosis (**Figure** **4**A,B).^[^ ^63^ ^]^ Another approach is to regulate iron‐associated proteins. As a vital LIP for Fe^2+^ storage, ferrtin degradation was considered an effective way to elevate intracellular free Fe^2+^ levels. Zhu et al.^[^ ^64^ ^]^ fabricated a ferritin‐hijacking nanoparticle (Ce6‐PEG‐HKN15). With the ferritin‐homing of HKN15 peptide, the photosensitizer chlorin e6 (Ce6) can specifically accumulate around ferritin. Under laser irradiation, the activated Ce6 in nanoparticles potently destroyed ferritin with the generated ROS and released Fe^2+^ for ferroptosis.

Recent studies have shown that ferroptotic cells can release DAMPs, thus initiating efficient antitumor immune responses. Yang's group constructed biomimetic Fe_3_O_4_ nanoparticles (Fe_3_O_4_‐SAS@PLT) with sulfasalazine (SAS) loading for enhanced ferroptosis.^[^ ^63^ ^]^ The study revealed that the nanoplatform‐mediated ferroptosis could not only induce tumor‐specific immune response but also efficiently repolarize macrophages from immunosuppressive M2 phenotype to antitumor M1 phenotype (Figure 4C,D). The nanomedicine significantly improved the efficacy of programmed cell death 1 (PD‐1) immune checkpoint blockade therapy and achieved continuous tumor elimination (Figure 4E,F). Hsieh et al.^[^ ^65^ ^]^ demonstrated that zero‐valent‐iron nanoparticles (ZVI‐NP) could also induce ferroptotic cancer cell death and reprogram the immunosuppressive microenvironment. In addition, Kim et al.^[^ ^66^ ^]^ found that ferumoxytol‐mediated ferroptosis could enhance the antitumor activity of natural killer (NK) cells.

In short, upregulation of intracellular Fe^2+^ level can efficiently kill tumor cells, not only by the Fenton reaction‐mediated ferroptosis, but by TME remodeling.

## 4 Characteristics of Cu^+^

### 4.1 Physicochemical Properties and Physiological Functions of Copper

Copper (element symbol: Cu) is a redox‐active transition metal element with the atomic number 29, presenting a reduced (Cu^+^) state or an oxidized (Cu^2+^) state. As the second important trace element in the human body, copper has a concentration of between 100 and 150 mg. Copper is a structural and catalytic cofactor of multiple enzymes, and participates in important life processes such as energy metabolism, antioxidant, neurotransmitter synthesis, and iron metabolism.^[^ ^67^ ^]^ In the cell, copper is involved in the functions of many copper‐dependent proteins, including oxidoreductases, monooxygenase and superoxide dismutase (SOD), transcriptional regulators, and chaperones.^[^ ^68^ ^]^ Once intracellular Cu^+^ overload, it can be oxidized and generate ▪OH, which can damage proteins, nucleic acids, and lipids, and interfere with the synthesis of iron–sulfur clusters that are essential for the activity of a number of important cellular enzymes.^[^ ^69^ ^]^ Since accumulation of intracellular copper is toxic, it is important to maintain copper homeostasis.

The concentration of intracellular Cu is tightly controlled by copper transporters (CTRs) and Cu chaperones. Since only Cu^+^ is transportable, extracellular Cu^2+^ that is combined with DMT1 should be reduced to Cu^+^ before cellular entry.^[^ ^70^ ^]^ CTRs are responsible for the influx of Cu^+^ across cell membranes, while ATPase copper‐transporting alpha (ATP7A) and ATPase copper‐transporting beta (ATP7B) are required for copper delivery to the secretory pathway and efflux of excess copper from the cell, respectively.^[^ ^71^ ^]^ Moreover, metallochaperones (e.g., antioxidant 1 copper chaperone, ATOX1) combine with free copper and facilitate its transport to, and incorporation into, important target proteins.^[^ ^72^ ^]^

### 4.2 Antitumor Mechanisms of Cu^+^ Dyshomeostasis‐Based Nanomedicines

It is evident that copper concentration is at a relatively higher level in tumor tissues than in normal tissues, contributing to tumor cell proliferation, angiogenesis, and metastasis.^[^ ^73^ ^]^ The biological effect of copper ions is U‐shaped (nonlinear), where high or low copper ion concentrations are conducive to cell death.^[^ ^74^ ^]^ Consequently, regulating intracellular copper ion concentration (Cu depletion and Cu supplementation) has emerged as an attractive novel target for tumor therapy. In this section, we will discuss the vital role of Cu depletion and Cu supplementation in tumor therapy and possible synergistic therapies.

#### 4.2.1 Cu Depletion‐Mediated Antitumor Effect

As mentioned above, Cu ions are involved in the structure and activity of Cu‐related proteins or enzymes, which are vital for tumor survival and development. So, Cu deficiency leads to impaired function of copper‐binding enzymes and deficiency of copper‐related proteins. For example, it was shown that all processes leading to cellular copper depletion result in cytochrome C oxidase deficiency due to the dependence of the respiratory chain complex on Cu as a cofactor.^[^ ^75^ ^]^ In view of this, reducing intracellular Cu levels would be a promising strategy for tumor therapy. To date, several small molecule drugs which either sequestrate copper ions from the cells or interfere with Cu transport, have been developed and applied in Cu metabolic dysfunction‐related diseases such as Wilson's disease by downregulating Cu level. Preclinical studies have shown that copper depletion therapy inhibits tumor growth, overcomes drug resistance such as Pt drugs, and potentiates antitumor immunotherapy via downregulation of programmed cell death ligand 1 (PD‐L1) expression.^[^ ^76^ ^]^ However, some unavoidable side effects and systemic adverse reactions were found during the treatment, such as skin rash, optic neuritis, etc., which may be due to the nonselective removal of copper ions from the body by copper chelators.^[^ ^77^ ^]^ Therefore, tumor‐targeted delivery of copper chelators is necessary for copper depletion therapy.

**Figure 5:**
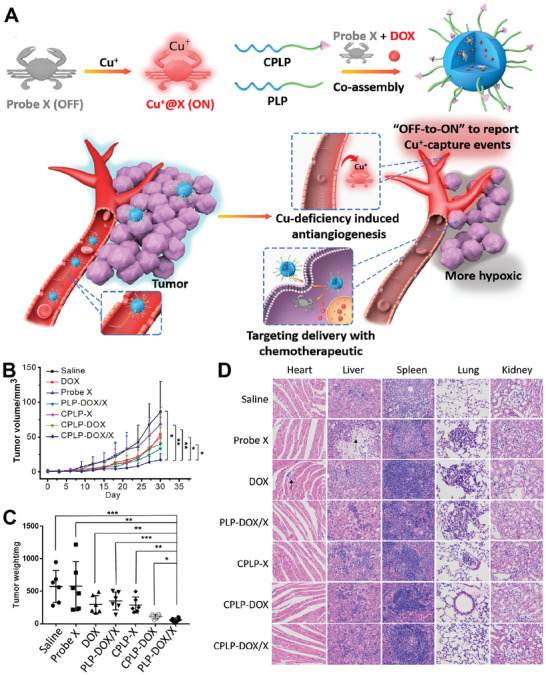
Copper chelator‐loaded nanomedicines for tumor therapy. A) Illustration of the co‐delivery of Cu^+^ chelator and chemotherapeutics as a new strategy for tumor theranostic. B) Tumor growth curve and C) Tumor weight of different groups. D) Histochemistry analysis of heart, liver, spleen, lung, and kidney sections stained with hematoxylin and eosin (H&E). Reproduced with permission.^[^ ^79^ ^]^ Copyright 2020, Elsevier.

With the advantages of tumor targeting and controllable drug release of nanomedicines, copper chelators are usually loaded into nanoparticles to avoid systematic toxicity. Cui and co‐workers developed a polymer Cu‐depleting nanoplatform (CDN) to deplete copper in the tumor.^[^ ^78^ ^]^ They revealed that Cu depletion by CDN resulted in a combination of energy and nutrient deficiency, as well as elevated oxidative stress and mitochondrial membrane rupture, which all contributed to the apoptosis of tumor cells. In vivo experiments showed that no cumulative and acute toxicity was observed after CDNs treatment, proving the safety of the nanomedicine. Additionally, Jiang's group co‐loaded DOX and a Cu^+^ Probe X (both as the chelator and tracer) into a micellar system built from a body‐friendly polypeptide amphiphile (**Figure** **5**A).^[^ ^79^ ^]^ Results demonstrated that the combination of Cu deficiency‐induced anti‐angiogenesis and DOX chemotherapy showed stronger tumor suppression and lower systematic toxicity (Figure 5B–D).

#### 4.2.2 Cu Supplementation‐Based Nanomedicines for Tumor Therapy

As previously mentioned, Cu overload is another effective way to kill tumor cells due to redox ability and structural and catalytic role in various proteins and enzymes. To date, the main approaches for Cu overload are copper ionophores, copper complexes, and Cu‐containing nanoparticles, where nanomaterials act as exogenous Cu^+^ or Cu^2+^ storage. The antitumor mechanisms of Cu‐based nanomedicines are attributed to the properties of Cu and the specific environment of tumor cells and tissues. The details are summarized as follows.

##### Fenton‐Like Reaction‐Mediated Cell Death

In tumor cells, a high concentration of glutathione (GSH) is conducive to the conversion of Cu^2+^ to Cu^+^. The redox‐active Cu^+^ catalyzes the Fenton‐like reaction, leading to the conversion of H_2_O_2_ to ▪OH. To one's excitement, the Cu^+^‐mediated Fenton‐like reaction can react in a wider pH range with a higher reaction rate (1 × 10^4^ M^−1^ S^−1^), reaching ≈ a 160‐fold increase over that of Fe^2+^.^[^ ^80^ ^]^ The Cu‐based catalytic reaction can be given in Equations (2) and (3).

(2) $$Cu^{2+}+\mathrm{GSH}\to Cu^{+}+\mathrm{GSSG}$$

(3) $$Cu^{+}+H_{2}O_{2}\to Cu^{2+}+•OH+OH^{-}$$

The abundant highly toxic ▪OH makes the Cu‐based nanomedicine a drug candidate for tumor therapy. However, the insufficient intracellular H_2_O_2_ limits the antitumor ability of Cu‐based nanomedicines. To amplify the oxidative stress by Cu^+^‐mediated Fenton‐like reaction, Lee et al.^[^ ^81^ ^]^ develop a hybrid polymer micelle (CuAS‐PM) integrated with a Cu^2+^‐arsenite (CuAS, Cu(HAsO_3_)) complex through a metal‐catechol chelation‐based approach. In the study, elevation of H_2_O_2_ levels in cancer cells by arsenic trioxide (ATO) via glutathione peroxidase (GPx) inhibition boosted Cu^+^‐mediated oxidative damage. Furthermore, radiotherapy can also increase intracellular H_2_O_2_, amplifying the oxidative stress, and indicating a potential synergistic antitumor effect.^[^ ^82^ ^]^

A high concentration of GSH is important to maintain redox homeostasis in the tumor cell, averting ROS damage. Hence, depletion of GSH may be beneficial for Cu^+^‐mediated Fenton‐like reaction. Lin's group synthesized self‐assembled Cu‐Cys NPs by a simple chemical coordination process between Cu^2+^ and the sulfhydryl groups of L‐cysteine in an alkaline solution.^[^ ^83^ ^]^ The study showed that a decrease in intracellular GSH and increased ROS generation by the Cu‐Cys NPs selectively killed cancer cells without obvious systemic toxicity. A combination of Cu^+^‐induced cytotoxicity and Ce6‐mediated sonodynamic therapy showed a synergistic antitumor effect.^[^ ^84^ ^]^ Due to the Cu^2+^‐dependent antitumor activity of disulfiram (DSF), Cu^2+^‐doped, DSF‐loaded hollow mesoporous silica nanoparticles (DSF@PEG/Cu‐HMSNs) were constructed for the rapid release of Cu^2+^ and DSF, exerting synergistic antitumor effect by Cu^+^‐mediated ROS generation and DSF‐mediated toxic CuET generation.^[^ ^85^ ^]^ Recently, Pei's group and Wang's group^[^ ^86^ ^]^ demonstrated that the synergistic effect of Cu^+^‐catalyzed generation of ▪OH and disulfide bonds‐mediated depletion of GSH by Cu‐based nanomedicines overcame chemoresistance by systemically disrupting dynamically balanced cellular redox homeostasis.

##### Cuproptosis and Cu^+^‐Mediated Ferroptosis and Pyroptosis

Cuproptosis is a mode of cell death induced by copper ion accumulation. According to the research reported in *Science*, increased intracellular Cu^+^ from Cu^2+^ reduction by mitochondrial ferredoxin 1 (FDX1) activates the aggregation of lipoylated mitochondrial proteins (e.g., dihydrolipoamide S‐acetyltransferase, DLAT) catalyzed by FDX1 and the destabilization of Fe–S cluster proteins, leading to proteotoxic stress and ultimately cell death.^[^ ^87^ ^]^ Endogenous intracellular GSH inhibits cuproptosis by functioning as a thiol‐containing copper chelator, the reduction of which can also lead to an increase in intracellular copper concentrations and cell death. For example, a study from Pan's group constructed the GOx@[Cu(tz)] nanomedicine for enhanced Cu^+^‐mediated cuproptosis. With the depletion of glucose and GSH, the Cu^+^ from the disassembled nanoparticles was more efficacious in binding to DLAT, producing aggregation of lipoylated DLAT, and resulting in GOx@[Cu(tz)]‐mediated cuproptosis.^[^ ^88^ ^]^ In another study, Zhao and coworkers developed a metal‐phenolic network nanoplatform (Cu–GA NPs) through self‐assembly of Cu^2+^ and gallic acid (Cu–GA).^[^ ^89^ ^]^ After internalization in tumor cells, Cu‐GA NPs were rapidly dissociated by GSH, leading to the release of GA and Cu^2+^, both of which can further consume GSH to reduce unwanted ROS consumption and copper ion chelation. The Cu^+^ converted by Cu^2+^ could not only catalyze the excess of H_2_O_2_ in the TME to produce plenty of ROS, leading to the apoptosis, but also bind to lipoylated DLAT, inducing its aggregation and downregulating Fe‐S cluster proteins, finally activating cuproptosis.

With the ability of ROS generation of Cu‐based nanomedicines via the Fenton‐like reaction, LPO in the cell membrane increased, followed by ferroptosis. As a copper ionophore, elesclomol preferentially chelates Cu^2+^ outside of cells and then rapidly and selectively transports the copper to mitochondria, where Cu^2+^ is reduced to Cu^+^, followed by subsequent ROS generation.^[^ ^90^ ^]^ Meanwhile, elesclomol promotes the degradation of the copper transporter ATP7A. Combinational treatment of elesclomol and copper leads to copper retention within mitochondria due to ATP7A loss, leading to ROS accumulation, which in turn promotes the degradation of SLC7A11, thus further enhancing oxidative stress and consequent ferroptosis.^[^ ^91^ ^]^ Another study from Liu's group demonstrated that exogenous copper could improve ferroptosis sensitivity by copper‐dependent autophagic degradation of glutathione‐dependent peroxidases 4 (GPX4).^[^ ^92^ ^]^ Besides ferroptosis, the studies from Yu's and Ge's group revealed that copper overload induced by Cu‐containing nanomedicines could also trigger pyroptosis via ROS generated by Cu^+^‐mediated Fenton‐like reaction.^[^ ^93^ ^]^

##### Modulation of Antitumor Immune Response

**Figure 6:**
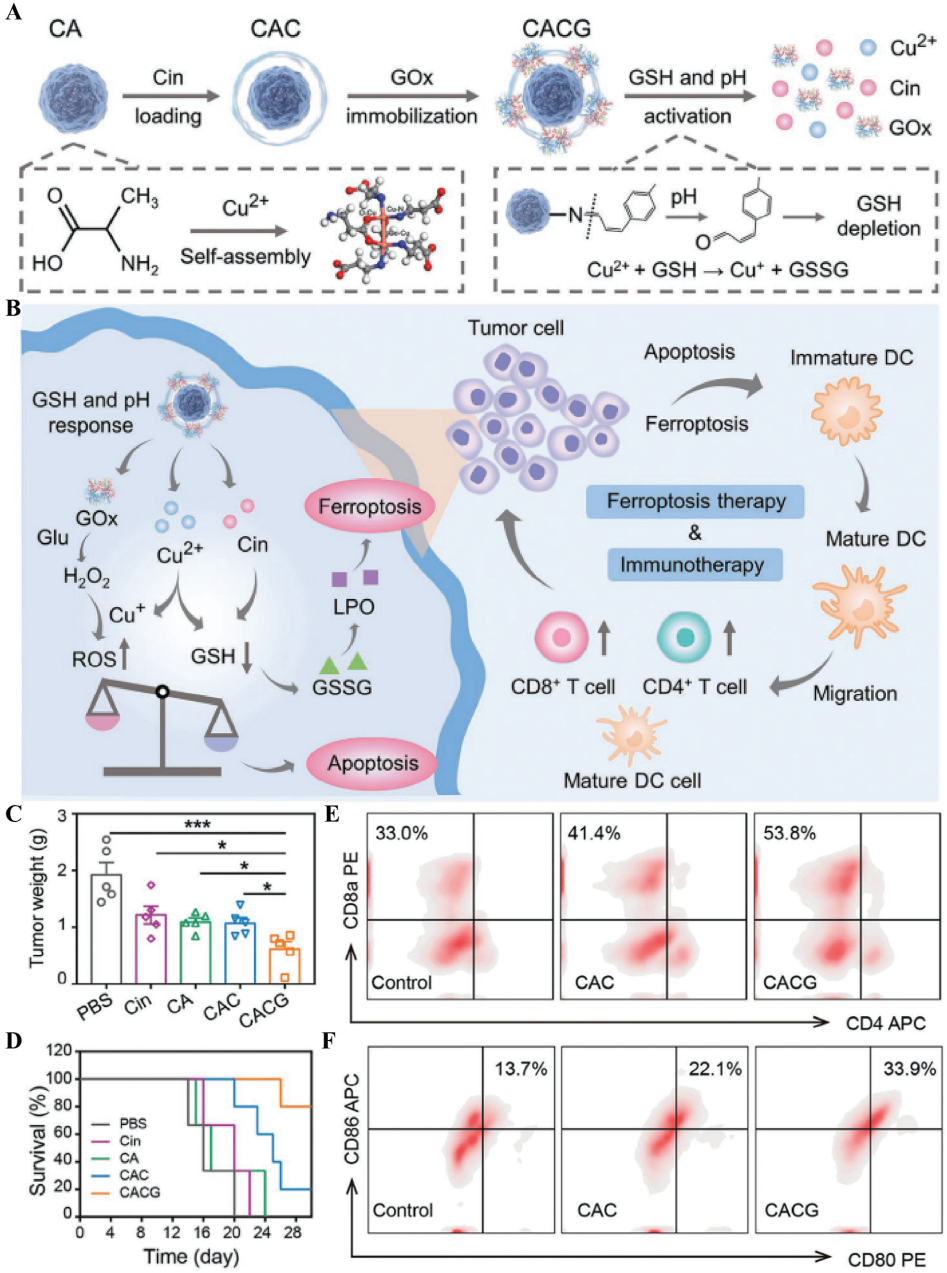
Cuproptosis‐mediated antitumor immune responses. A) Synthetic process of CACG nanoparticles. B) Illustration of ferroptosis/apoptosis‐mediated antitumor immune activation. C) Tumor weight and D) Survival rate in different groups. E) Flow cytometry analysis of the infiltration of CD8^+^ T cells in tumors with different treatments. F) Flow cytometry analysis of the DC maturation in lymph nodes of mice immunized with different treatments. Reproduced with permission.^[^ ^94^ ^]^ Copyright 2023, Wiley‐VCH GmbH.

Notably, mitochondrial dysfunction and ROS generation by Cu^+^ cause significant ICD, potentiating tumor immunotherapy with the assistance of PD‐L1 antibodies. Zhang's group demonstrated that increased H_2_O_2_ by glucose oxidase (GOx) and decreased GSH by cinnamaldehyde (Cin) and Cu^2+^ reduction not only disrupted redox homeostasis but enhanced ferroptosis, promoting DC maturation and cytotoxic CD8^+^ T cell‐mediated antitumor effect (**Figure** **6**).^[^ ^94^ ^]^ Insignificant pathological abnormalities in major organs and no significant change in blood routine analysis and biochemistry analysis of mice with different treatments demonstrated the negligible side effects and good biocompatibility of the constructed nanomedicines for biomedical application in vivo. Additionally, lactate depletion and H_2_O_2_ elevation by lactate oxidases helped Cu^+^‐induced ICD initiate the positive immune response effectively.^[^ ^95^ ^]^ Guo et al.^[^ ^96^ ^]^ further illustrated that the ROS‐sensitive nanoplatform (NP@ESCu) with co‐encapsulation of elesclomol and Cu not only could promote ICD‐induced DCs maturation and CD8^+^ T cell infiltration in tumor tissues but also reprogram the immunosuppressive TME via repolarization of tumor‐associated M2 phenotype macrophages, as well as inhibition of myeloid‐derived suppressor cells (MDSCs), ultimately enhancing anti‐tumor immune responses combining with αPD‐L1. The Cu‐doped polypyrrole nanozyme (CuP) with trienzyme‐like activities, including catalase (CAT), GPx, and peroxidase (POD) could also efficiently reverse immunosuppressive TME by overcoming tumor hypoxia and re‐educating tumor‐associated M2 macrophage.^[^ ^97^ ^]^

Collectively, Cu ions overload‐based nanomedicines can disrupt redox homeostasis via the Fenton‐like reaction, as well as induce cuproptosis, ferroptosis, and pyroptosis, promoting antitumor response and remodeling immunosuppressive TME.

## 5 Characteristics of Mn^2+^

### 5.1 Physicochemical Properties and Physiological Functions of Manganese

Manganese (element symbol: Mn) is a transition element with an atomic number of 25, and its valence states are 0, +2, +3, +4, +6, and +7. However, the most common forms of Mn found in living tissues are Mn^2+^ and Mn^3+^. In human beings, Mn is a nutritional inorganic trace element involved in multiple physiological processes, such as development, energy metabolism, antioxidant defenses, and immune function, due to its important role in the structure and activities of numerous metalloenzymes that are involved in a wide range of cellular processes.^[^ ^98^ ^]^ For example, as a Mn‐associated metalloenzyme, manganese superoxide dismutase (MnSOD) is a critical mitochondrial antioxidant and is responsible for the detoxification of superoxide radicals.^[^ ^99^ ^]^ Nevertheless, on account of good redox properties, Mn has the strong ability of ▪OH generation via the Fenton‐like reaction, increasing oxidative stress.^[^ ^100^ ^]^ In addition, Mn is a vital catalytic cofactor of the cyclic guanosine monophosphate‐adenosine monophosphate synthase (cGAS) protein, a DNA sensor to activate the host immune response.^[^ ^101^ ^]^

The essential yet toxic nature of Mn necessitates precise homeostatic mechanisms to maintain an appropriate level of intracellular Mn. Mn homeostasis in humans is regulated by manganese efflux pump (MntE) and metal transporters ZIP8 (SLC39A8), ZIP14 (SLC39A14), and ZnT10 (SLC30A10).^[^ ^102^ ^]^ In addition, Mn is also transported by iron transporters, since Mn possesses similar chemical properties to iron.^[^ ^103^ ^]^

### 5.2 Antitumor Mechanisms of Mn^2+^‐Related Nanomedicines

In recent years, Mn‐containing nanoparticles have been widely used as magnetic resonance imaging (MRI) T1 contrast agents for tumor diagnosis, and as carriers of antitumor drugs for tumor treatment.^[^ ^104^ ^]^ As a transition metal like Fe, Mn can also decompose H_2_O_2_ to produce ▪OH via the Fenton‐like reaction, for direct tumor killing. In addition, as an immune adjuvant, Mn^2+^ can also activate the cGAS‐stimulator of interferon genes (STING) signaling pathway to produce an immune response. Mn oxides and other Mn‐containing nanomedicines are commonly used for increasing intracellular Mn^2+^ concentration due to their multiple oxidation states, low cost and mature synthesis processes, and drug loading capacity. In this section, the detailed antitumor mechanisms of Mn‐related nanomedicines and the potential synergistic therapies will be discussed.

#### 5.2.1 Fenton‐Like Reaction‐Mediated Antitumor Effect and Intracellular Environment Regulation by Mn‐Containing Nanomedicines

Due to the relatively high concentration of GSH and acidic lysosomes in tumor cells, Mn‐containing nanomedicines in recent years have been used to elevate intracellular free Mn^2+^. After internalization, some Mn‐based nanoparticles can consume intracellular GSH, conducive to the ROS enrichment in the tumor. In addition, they can also catalyze H_2_O_2_ to produce oxygen (O_2_), thus alleviating hypoxia for enhancement of therapy sensitization such as radiotherapy and starvation therapy.^[^ ^105^ ^]^ With the redox property, released Mn^2+^ from degradative nanoparticles can produce •OH via the Fenton‐like reaction with H_2_O_2_, leading to ferroptosis. Wang et al.^[^ ^106^ ^]^ demonstrated that arginine‐rich manganese silicate nanobubbles (AMSNs) could induce ferroptosis via highly efficient GSH depletion and inactivation of GPX4. In addition, a manganese porphyrin‐based metal–organic framework (Mn‐MOF) constructed by Gan's group^[^ ^107^ ^]^ worked as a nanosensitizer to self‐supply O_2_ and decrease GSH for enhanced sonodynamic therapy and ferroptosis. The Mn‐based catalytic reactions are shown in Equations (4)–(6).

(4) $$MnO_{2}+GSH\to Mn^{2+}+GSSG$$

(5) $$MnO_{2}+H_{2}O_{2}+H^{+}\to Mn^{2+}+H_{2}O+O_{2}$$

(6) $$H_{2}O_{2}\overset{Mn^{2+}/HCO_{3}^{-}}{\to }•OH$$

#### 5.2.2 Activation of Innate and Adaptive Immune Responses and Remodeling of TME

**Figure 7:**
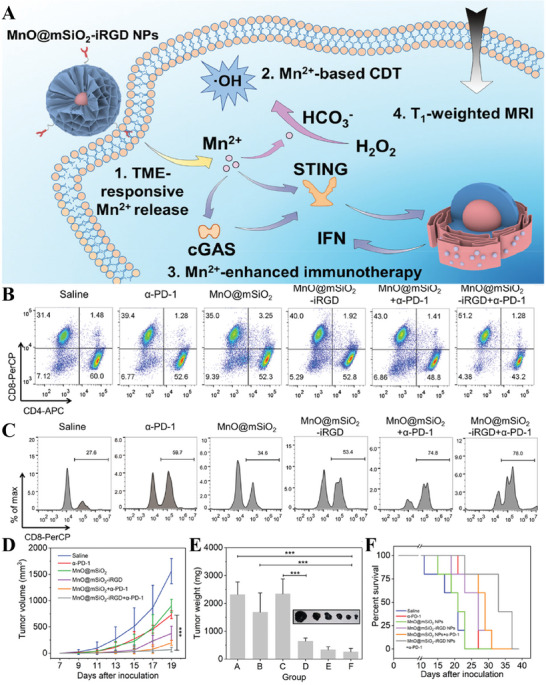
Mn containing nanomedicine (MnO@mSiO_2_‐iRGD NPs) for tumor therapy. A) Schematic illustration of antitumor mechanisms of Mn^2+^ containing nanomedicines. B) The frequency of CD8^+^ T cell and CD4^+^ T cell in the spleen possessed from mice in the differently treated group by flow cytometry (gated on CD3^+^). C) Tumor‐infiltrating CD8^+^ T cells of mice. D) Tumor volume changes, E) Tumor weight, and F) Survival rate in different groups. Reproduced with permission.^[^ ^111^ ^]^ Copyright 2022, American Chemical Society.

cGAS‐STING signaling pathway plays a crucial role in the innate immune response, in which cGAS is a DNA sensor. In the damaged tumor cell, tumor‐derived double‐stranded DNA will be released to the cytoplasm or outside cells. Upon binding with the exposed DNA, cGAS catalyzes ATP and guanosine triohosphte (GTP) to cyclic guanosine monophosphate‐adenosine monophosphate (cGAMP), which activates the ER adaptor protein STING and elicits an immune response.^[^ ^108^ ^]^ As the activator of cGAS, Mn^2+^ not only increases the catalytic ability of cGAS but also enhances the affinity of cGAMP and STING on the surface of ER, to give rise to interferon regulatory factor 3 (IRF3) phosphorylate and activate the nuclear factor‐κ‐gene binding (NF‐κB) pathway, promoting type‐I interferon (IFN) production and enhancing CD8^+^ T cell function.^[^ ^109^ ^]^ Co‐delivery of Mn^2+^ and STING agonists by a coordination nanoparticle dramatically augmented STING activation and amplified the type‐I IFN responses.^[^ ^7^ ^a]^ Synchronizing Mn^2+^ delivery with accumulated cytosolic DNA after radiotherapy or chemotherapy can promote the activation of the cGAS‐STING pathway, thereby enhancing radiotherapy or chemotherapy‐induced antitumor immunity.^[^ ^110^ ^]^ In addition, ICD triggered by Fenton‐like reaction‐induced ROS contributes to DAMPs release and DC maturation‐mediated antitumor immunity. For example, Sun et al.^[^ ^111^ ^]^ revealed that MnO@mSiO_2_‐iRGD NPs could be harnessed for Fenton‐like reaction‐induced ROS upregulation, cGAS‐STING pathway‐activated immunotherapy, highly eliciting cytotoxic T lymphocyte infiltration and tumor suppression combining with αPD‐1 (**Figure** **7**). MnO@mSiO_2_‐iRGD NPs NPs administration did not reduce body weight compared to those treated with saline or α‐PD‐1 for subcutaneous tumor models, confirming the safety of NPs. Zhao et al.^[^ ^112^ ^]^ designed a tumor cell membrane (CM)‐wrapping multienzyme‐mimic manganese oxide (MnOx) nanozyme termed CM@Mn. The study showed that TME‐responsive release of Mn^2+^ from CM@Mn could reverse immunosuppressive TME via oxidative damage‐mediated tumor cell apoptosis, and ICD and STING pathway‐mediated immune cell activation.

In a word, Mn^2+^‐related nanomedicines can not only directly kill tumor cells by Fenton‐like reaction‐induced ROS, but also activate innate and adaptive immune responses by both ROS‐induced ICD and activation of cGAS‐STING pathway.

## 6 Characteristics of Zn^2+^

### 6.1 Physicochemical Properties and Physiological Functions of Zinc

Zinc (element symbol: Zn) is a chemical element with the atomic number 30 and its common ionic form is Zn^2+^. Zinc is the second most abundant transition metal, containing 2–3 g of zinc in humans. The majority of Zn is cumulative inside cells. Intracellular Zn exists in three forms: 1) Tightly bounding to metalloenzymes, metalloproteins, and nucleoproteins, 2) Loosely binding with various proteins and amino acid ligands, and 3) Free Zn^2+^ ions.^[^ ^113^ ^]^

Zn^2+^ is known to be an intracellular second messenger, like Ca^2+^, related to various biological functions.^[^ ^114^ ^]^ Zn is necessary for the body's proper physiological functions, like normal growth, reproduction, DNA synthesis, cell division and gene expression, wound healing, and immune system augmentation of the body. At the cellular level, zinc ion is a cofactor of many enzymes and transcription factors, and serves as a structural ion, helping to stabilize the enzyme structure.

Due to the toxicity of excessive Zn^2+^ to cells, Zn levels are tightly regulated within cells. Intracellular Zn homeostasis is mainly regulated by metallothioneins (MTs) and two opposite zinc transporters (i.e., ZnT (SLC30s) and Zip (SLC39s) families) through the cytoplasm and cellular membranes.^[^ ^115^ ^]^ In addition, zinc also enters cells via other ion channels, such as Zn^2+^‐permeable voltage‐gated Ca^2+^ ion channels.^[^ ^116^ ^]^ The maintenance of zinc homeostasis is a requisite for normal physiological processes.

### 6.2 Antitumor Mechanisms of Zn^2+^‐Based Nanomedicines

Reports have revealed that excessive Zn^2+^ possesses antitumor ability in various tumors such as prostatic cancer and breast cancer.^[^ ^117^ ^]^ Zinc supplementation augments chemotherapy by restoring p53 function.^[^ ^118^ ^]^ Zinc ionophores (e.g., pyrithione) and Zn‐containing nanoparticles (e.g., zeolitic imidazolate framework (ZIF) nanoparticles) are frequently used for Zn^2+^‐based tumor therapy. The antitumor mechanisms of Zn^2+^‐based nanomedicines mainly include Zn^2+^ overload‐mediated mitochondrial dysfunction and subsequent antitumor immune responses. The detailed mechanisms are shown as follows.

#### 6.2.1 Zn^2+^ Overload‐Mediated Mitochondrial Dysfunction for Cell Death

Different from Fe^2+^, Cu^+^, or Mn^2+^, Zn^2+^ breaks down the antioxidant system and increases endogenous ROS via inhibiting the glutathione reductase (GR) and thioredoxin reductase (TrxR) activities, enhancing ROS‐mediated therapeutic effects of tumor cells.^[^ ^119^ ^]^ Also, Zn^2+^ leads to mitochondrial dysfunction by inhibiting their oxidative respiration, thus decreasing intracellular ATP levels. Moreover, Wu et al.^[^ ^120^ ^]^ illustrated that Zn^2+^ released from ZIF‐8 inhibited glycolysis through reduction of NAD^+^ and inactivation of GAPDH, leading to ATP decrease and remarkable systematic energy exhaustion‐mediated cell death with Zn^2+^‐activated DNAzymes that specifically cleave GLUT1 mRNA.

However, some other studies demonstrated that ZIF‐8 NPs can induce autophagy to promote the survival of cancer cells via Zn^2+^‐stimulated mitochondrial ROS.^[^ ^121^ ^]^ Autophagy inhibition by an autophagic inhibitor (chloroquine) or ATG5 knockdown significantly boosted the ZIF‐8‐elicited antitumor effect.^[^ ^122^ ^]^ Nevertheless, another study from Li's group^[^ ^123^ ^]^ showed that ZIF‐8 could sensitize tumor cells to chemotherapy by switching cytoprotection to death and promoting autophagy induced by doxorubicin, which would be enhanced by combination of autophagic inducer rapamycin.

#### 6.2.2 Zn^2+^‐Mediated Activation of Innate and Adaptive Immune Responses

**Figure 8:**
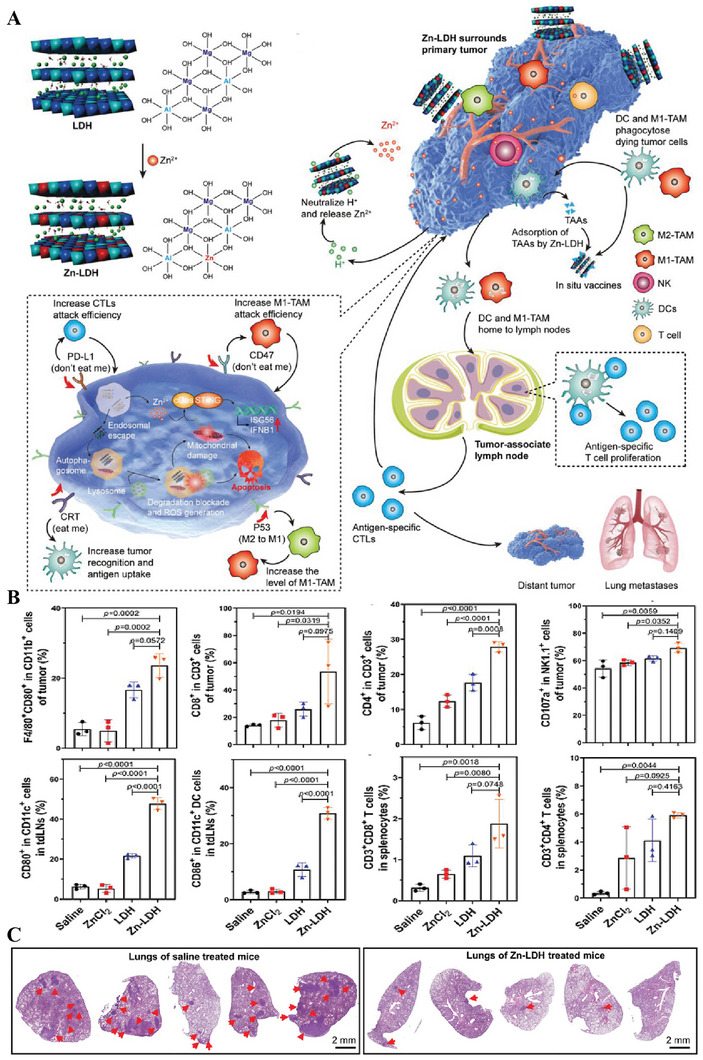
Zn^2+^ containing nanomedicines for tumor therapy. A) Released Zn^2+^ from Zn‐LDH nanomedicines activated the cGAS‐STING signaling pathway to induce ICD‐mediated antitumor immunity. B) Levels of different immune cells in tumor and tdLNs. C) H&E staining analysis of the lung metastasis of mice. Reproduced with permission.^[^ ^126^ ^]^ Copyright 2022, Wiley‐VCH GmbH.

Because of the ability of abundant ROS production, Zhang's group^[^ ^124^ ^]^ found that Zn^2+^ release from Zn‐Fu MNs could induce ferroptosis by inhibiting the mitochondrial electron transport chain, leading to a violent ICD burst in tumor cells with 5‐Fu chemotherapy and effective remodeling of immunosuppressive tumor microenvironment. Another study from Lin's group^[^ ^125^ ^]^ showed that Zn^2+^ released from ZIF‐8 NPs elicited pyroptosis by a sudden surge in ions and intracellular osmolarity‐mediated activation of caspase‐1/GSDMD‐dependent pyroptosis pathway, which activated antitumor immunity and reprogrammed immunosuppressive TME. In addition, Zhang et al. demonstrated that the released Zn^2+^ from Zn‐LDH nanomedicines could also induce ICD by activating the cGAS‐STING signaling pathway, promoting DC maturation and activating antitumor immune responses of cytotoxic CD8^+^ T cells (**Figure** **8**).^[^ ^126^ ^]^ No evident changes in body weight and the levels of alanine transaminase (ALT) and aspartate aminotransferase (AST) in different groups in melanoma or breast cancer models indicated the safety of metalloimmunotherapy. Ding et al.^[^ ^127^ ^]^ constructed a zinc‐organometallic framework vaccine (ZPM@OVA‐CpG) by self‐assembly, and illustrated that Zn^2+^ released from the vaccine significantly enhanced the infiltration and killing of CD8^+^ T cells by up‐regulation of the matrix metalloproteinase‐2 activity and degradation of tumor extracellular matrix, besides the activation of cGAS‐STING signaling pathway.

Taken together, Zn^2+^‐based nanomedicines can elicit ROS‐mediated cell death via mitochondrial dysfunction and induce ICD via a variety of signal pathways to enhance tumor immunotherapy.

## 7 Conclusions and Future Perspectives

With the approval of metallodrugs like Pt drugs for tumor therapy and a deepening understanding of the important role of intracellular various ions in tumor development and immunity, some novel therapies based on metal ion regulation have emerged such as cancer metalloimmunotherapy and ion‐interfering therapy. Recently, great progress has been made in the research of various metal ion regulation‐based nanomedicines for tumor therapy. The increasing amounts of compounds (e.g., curcumin, deferoxamine, disulfiram) with metal ion regulation ability have been developed for tumor therapy and some nanomaterials (e.g., liposomes, and polymer nanoparticles) have been approved for drug carriers in clinics. In addition, self‐therapeutic metal‐based nanoagents demonstrated promising antitumor effects in preclinical studies. For example, Rizzolio's group^[^ ^128^ ^]^ demonstrated that metal‐based nanomaterials cobalt hydroxide nanosheets showed excellent potency, comparable to the FDA‐approved cisplatin drug to kill ovarian cancer cells and low toxicity on normal cells. The antitumor activity of Fe_3_O_4_ nanoparticles, CaCO_3_ nanoparticles, and CaP nanoparticles may broaden their clinical application. Therefore, metal ion regulation‐based nanomedicines have great potential for tumor therapy and clinical transition and it is essential to understand and master the regulation mechanisms of ions in tumor cells and normal cells.

This review summarizes the physiological functions and homeostasis regulation of the five common metal ions (Ca^2+^, Fe^2+^, Cu^+^, Mn^2+^, and Zn^2+^) in human beings to help researchers develop more different approaches to metal ion regulation. After that, the current research progresses on antitumor mechanisms, and possible synergistic antitumor therapies of metal ion regulation‐based nanomedicines are discussed. From the summary, we can see that these metal ions can induce cell death in multiple ways such as apoptosis, pyroptosis, ferroptosis, and cuproptosis, which is conducive to the search for more improved combination strategies and more useful metal ions for tumor therapy. For example, since various metal ions are involved in virus replication and clearance via nutrient supply and immune regulation, whether the combination of metal ion regulation‐based nanomedicines and oncolytic virotherapy exert a synergistic antitumor effect? In addition, this review will provide a reference for research on the antitumor effects and mechanisms of other endogenous metals (e.g., magnesium ions, and selenium). Therefore, metal ion regulation‐mediated nanomedicines offer an alternative choice for tumor therapy.

However, the clinical translational application of metal ion regulation‐based nanomedicines for tumor therapy still faces some problems. First, although many antitumor mechanisms of metal ion regulation‐based nanomedicines have been reported, most of them are validated only in several cell lines, which can not represent the complex tumor cells in tumor tissues from patients at different stages. So, more research has to be done in more cell lines, organoids, and animal models to clarify the antitumor mechanisms and effects of metal ion regulation‐based nanomedicines.

Second, the understanding of how various ions are integrated into the cellular milieu is important to guide the combination of various types of ion interference therapy or other approaches to tumor therapy. The effects of different ions on immune cells and tumor cells need to be further studied.

Finally, the biological safety of nanomedicines in vivo is still the biggest challenge for their clinical applications. At present, most studies only examine the short‐term toxicity of nanomedicines and the corresponding structural damage to main organs. Therefore, before their clinical transformation, further research is required for in vivo behavior, metabolic pathways, long‐term toxicity, dose‐dependent toxicity, and physiological function changes of main organs. And, large animal studies are also necessary for the translation from bench to clinic.

## Conflict of Interest

The authors declare no conflict of interest.
